# Crystal structure of *cyclo*-bis­(μ_4_-2,2-di­allyl­malonato-κ^6^ *O* ^1^,*O* ^3^:*O* ^3^:*O* ^1′^,*O* ^3′^:*O* ^1′^)tetra­kis­(triphenyl­phosphane-κ*P*)tetra­silver(I)

**DOI:** 10.1107/S1600536814019394

**Published:** 2014-09-10

**Authors:** Peter Frenzel, Alexander Jakob, Dieter Schaarschmidt, Tobias Rüffer, Heinrich Lang

**Affiliations:** a Technische Universität Chemnitz, Faculty of Natural Sciences, Institute of Chemistry, Inorganic Chemistry, 09107 Chemnitz, Germany

**Keywords:** crystal structure, silver(I), malonate ligand, phosphane ligand, Ag_4_O_8_P_4_ core

## Abstract

In the tetra­nuclear mol­ecule of the title compound, [Ag_4_(C_9_H_10_O_4_)_2_(C_18_H_15_P)_4_], the Ag^I^ ion is coordinated by one P and three O atoms in a considerably distorted tetra­hedral environment. The two 2,2-di­allyl­malonate anions bridge four Ag^I^ ions in a μ_4_-(κ^6^ *O* ^1^,*O* ^3^:*O* ^3^:*O* ^1′^,*O* ^3′^:*O* ^1′^) mode, setting up an Ag_4_O_8_P_4_ core (point group symmetry -4..) of corner-sharing tetra­hedra. The shortest intra­molecular Ag⋯Ag distance of 3.9510 (3) Å reveals that no direct *d* ^10^⋯*d* ^10^ inter­actions are present. Four weak intra­molecular C—H⋯O hydrogen bonds are observed in the crystal structure of the title compound, which most likely stabilize the tetra­nuclear silver core.

## Abstract 2

In the title compound, the silver(I) ions are coordinated by four tri­phenyl­phosphane ligands and two 2,2-di­allyl­malonate anions in a μ_4_-(κ^6^ *O* ^1^,*O* ^3^:*O* ^3^:*O* ^1′^,*O* ^3′^:*O* ^1′^) mode, setting up an Ag_4_O_8_P_4_ core.

## Chemical context   

Silver(I) carboxyl­ates of general type [AgO_2_C*R*]_*n*_ (*n* is the degree of aggregation) are of inter­est due to their versatile structures in the solid state and in solution, their synthetic methodologies and their manifold reaction behavior (see, for example: Schliebe *et al.*, 2013 ▶; Jahn *et al.*, 2010 ▶; Wang *et al.*, 2008 ▶; Fernández *et al.*, 2007 ▶; Olson *et al.*, 2006 ▶; Szymańska *et al.*, 2007 ▶). These metal-organic complexes are of importance not only in the field of basic research but also in multipurpose applications including, for example, metallization processes for micro- and nano-structured new materials in electronic systems and devices (*e.g*. using chemical vapour deposition, CVD), since silver possesses the highest electrical conductivity of any element (Jakob *et al.*, 2010 ▶; Lang & Dietrich, 2013 ▶), catalytic processes (Steffan *et al.*, 2009 ▶) and their use in biological studies (Djokić, 2008 ▶; Zhu *et al.*, 2003 ▶).
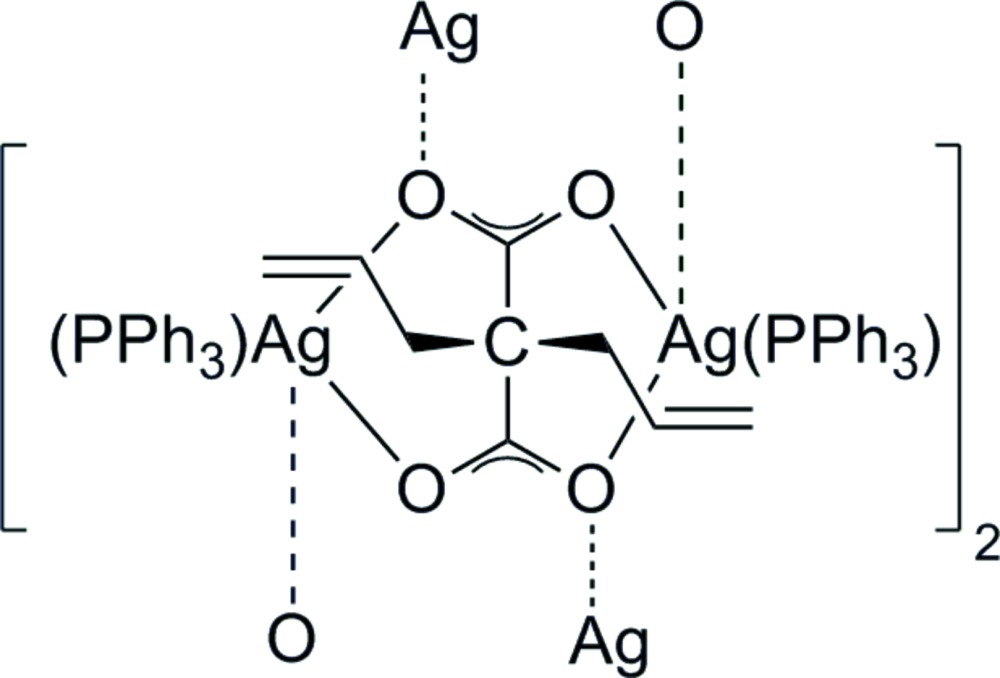


The CVD process requires metal precursors possessing high vapour pressures. On a mol­ecular level this is typically achieved by designing low aggregated metal compounds. In the case of silver, this can be realized by the use of phosphanes as a Lewis base; however, the concomitant increase of the mol­ecular weight of the transition metal complex may decrease its vapour pressure. Circumventing this difficulty, we have investigated the use of olefines as ligands for silver(I) carboxyl­ates, in which the olefin is covalently bonded to the carboxyl­ate. In the context of this approach, the title compound [{(Ph_3_P)Ag}_4_{(O_2_C)_2_C(CH_2_CH=CH_2_)_2_}_2_], (I), was obtained by the reaction of the silver salt of 2,2-di­allyl­malonic acid with tri­phenyl­phosphane.

## Structural commentary   

**Figure 1:**
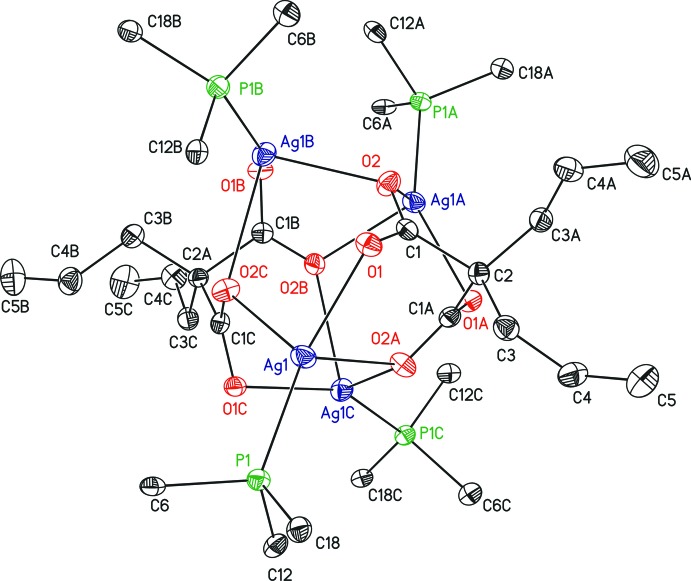
The Ag_4_O_8_P_4_ core of the title compound with surrounding atoms. Displacement ellipsoids are displayed at the 50% probability level. The carbon atoms of the phenyl substituents except the *ipso*-carbon atoms and all hydrogen atoms have been omitted for clarity. [Symmetry codes: (A) –*x* + 1, –*y* + 1, *z*; (B) *y*, –*x* + 1, –*z* + 2; (C) –*y* + 1, *x*, –*z* + 2.]

The asymmetric unit of (I) contains one quarter of the mol­ecule which is completed by application of a fourfold screw axis as the symmetry element. The resulting tetra­nuclear silver core is decorated by four tri­phenyl­phosphane ligands, whereby the metal ions are bridged by two 2,2-di­allyl­malonate anions in a μ_4_-(κ^6^ *O* ^1^,*O* ^3^:*O* ^3^:*O* ^1′^,*O* ^3′^:*O* ^1′^) mode (Fig. 1 ▶). There is no example in the literature of a transition metal malonate displaying this type of coordination. The environment around silver, set up by one phospho­rus and three oxygen atoms, is best described as distorted tetra­hedral. Ag1 is oriented slightly above the plane of O1, P1 and O2^ii^ [distance 0.2911 (10) Å], which is supported by the respective bond angles around Ag1 (Table 1 ▶) summing up to 354.3°. The O—Ag1—P1 angles are substanti­ally larger than the O—Ag1—O angles, which may be attributed to the chelating coordination of the malonate ligands and the bulkiness of the tri­phenyl­phosphane ligand. The Ag—O bond lengths are more than 0.2 Å shorter for the two oxygen atoms of the aforementioned plane than for the third apical oxygen atom (Table 1 ▶). However, the values are in the expected range for Ag—O bonds in silver carboxyl­ates.

**Figure 2:**
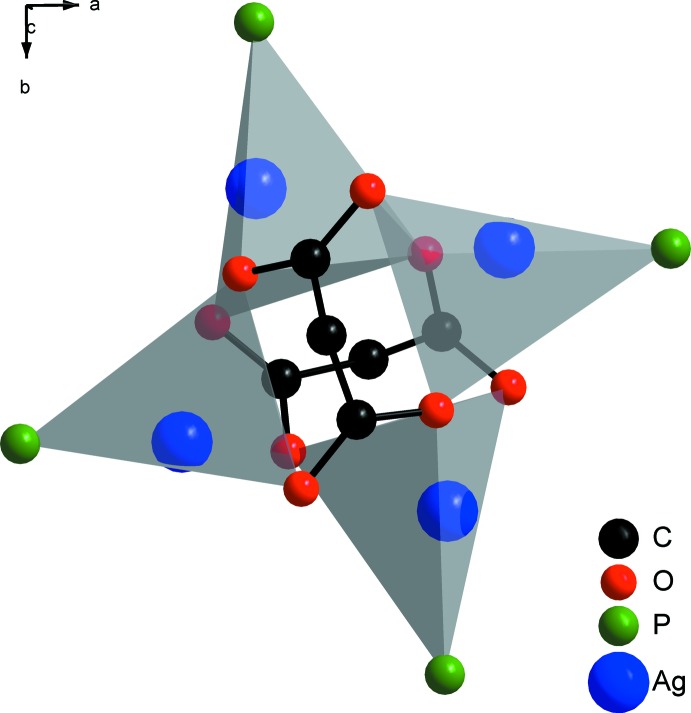
Structure fragment showing the cyclic corner-sharing arrangement of the AgO_3_P polyhedra giving the tetra­nuclear silver core of composition Ag_4_O_8_P_4_.

The cyclic corner-sharing arrangement of the described O_3_P tetra­hedra gives the tetra­nuclear structure of (I) (Fig. 2 ▶). The four silver ions are oriented in a butterfly-like arrangement, which delimits the title compound from Ag_4_O_4_ heterocubanes (Jakob *et al.*, 2011 ▶; Zhang *et al.*, 2008 ▶, Kühnert *et al.*, 2007 ▶) in which the four silver ions form a tetra­hedron. In contrast, there are some similarities with [bis­(1,8-naphthalenedi­carboxyl­ato)][tetra­kis­(tri­phenyl­phosphane)silver(I)] (van der Ploeg *et al.*, 1979 ▶); however, in the structure of this compound one silver ion is penta­coordinated.

## Supra­molecular features   

Four weak intra­molecular C—H⋯O hydrogen bonds (Steiner, 2002 ▶) are observed in the crystal structure of (I) (Table 2 ▶), which most likely stabilize the silver core.

**Figure 3:**
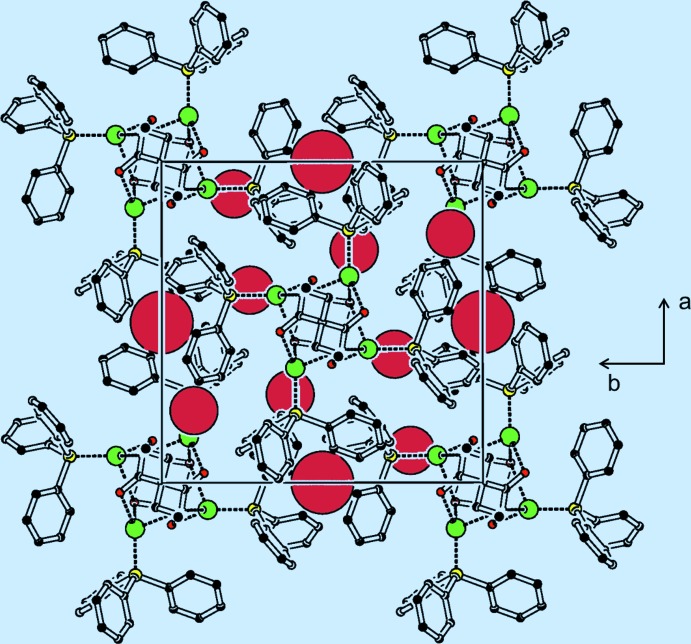
Packing diagram of the title compound along the *c* axis; voids in the structure are represented by red spheres [drawn using the CAVITYPLOT routine in *PLATON* (Spek, 2009 ▶)]. The hydrogen atoms have been omitted for clarity. Colour code: black (C), red (O), yellow (P), green (Ag).

In contrast to iridium and platinum complexes of 2,2-diallylmalonic acid and derivatives thereof, the C=C double bond does not coordinate the transition metal in (I). Furthermore, no obvious π–π stacking inter­actions are observed between the allyl and the phenyl substituents. Therefore, the packing seems to be dominated by dispersion forces (Fig. 3 ▶).

## Database survey   

2,2-Di­allyl­malonic acid and derivatives thereof have only been used as ligands in four mononuclear platinum and one iridium complex, in which coordination of the transition metal occurs either through (*O*,*O*′)-, (*O*,alkene)- or (alkene,alkene′)-chelation (Berthon-Gelloz *et al.*, 2007 ▶; Makino *et al.*, 2004 ▶; Jung *et al.*, 1999 ▶; Lee *et al.*, 1999 ▶). To the best of our knowledge, no di­allyl­malonate silver(I) compounds have been described in the literature so far.

## Synthesis and crystallization   

Complex [{(Ph_3_P)Ag}_4_{(O_2_C)_2_C(CH_2_CH=CH_2_)_2_}_2_] was prepared by the addition of PPh_3_ (132 mg, 0.503 mmol) to a suspension of [(AgO_2_C)_2_C(CH_2_CH=CH_2_)_2_] (100 mg, 0.251 mmol) in di­chloro­methane (30 ml) at 273 K. After stirring for 2 h at this temperature, the reaction mixture was filtered through a pad of celite. Afterwards, all volatiles were removed in oil-pump vacuum, and (I) was obtained as a pale-grey solid. Colourless crystals of (I) were obtained by solvent diffusion of a chloro­form solution of (I) against pentane at ambient temperature. Yield: 230 mg (0.125 mmol, 99% based on [(AgO_2_C)_2_C(CH_2_CH=CH_2_)_2_]).

Analysis calculated for C_90_H_80_Ag_4_O_8_P_4_ (1844.96): C 58.59, H 4.37. Found: C 58.53, H 4.34. ^1^H NMR (500 MHz, CDCl_3_, 298 K, ppm): δ = 2.79 (*d*, 8H, ^3^ *J*_HH_ = 6.5 Hz, C*H*_2_CH=CH_2_), 4.97 (*d*, 4H, ^3^ *J*_HH_ = 10.2 Hz, CH_2_CH=C*H*_2_), 5.03 (*d*, 4H, ^3^ *J*_HH_ = 17.1 Hz, CH_2_CH=C*H*_2_), 5.90 (*m*, 4H, CH_2_C*H*=CH_2_), 7.30–7.51 (*m*, 60H, C_6_H_5_). ^31^P{^1^H} NMR (203 MHz, CDCl_3_, 298 K, ppm): δ = 15.7 (*d*, ^1^ *J*_AgP_ = 680 Hz). IR (KBr, cm^−1^): *ν* = 1637 (*w*, C=C), 1559 (*vs*, C=O), 1440 (*vs*, P—Ph), 692 (*vs*), 521 (*vs*).

## Refinement   

Crystal data, data collection and structure refinement details are summarized in Table 3 ▶. C-bonded H atoms were placed in calculated positions and constrained to ride on their parent atoms, with *U*_iso_(H) = 1.2*U*_eq_(C) and a C—H distance of 0.93 Å for aromatic and vinylic as well as 0.97 Å for methyl­ene protons. The unit cell contains two voids of 34(1.4) Å^3^. Void volume calculation using the SQUEEZE routine in *PLATON* (Spek, 2009 ▶) gives a total electron count in the voids per cell of 3 e^−^ Å^−3^ suggesting that no solvent mol­ecules occupy these voids. The Flack parameter is −0.051 (9); however, this ambiguity is resolved as the Flack parameter of the inverted structure is calculated to 1.052 (9). This indicates that the original absolute structure has been assigned correctly.

## Supplementary Material

Crystal structure: contains datablock(s) I. DOI: 10.1107/S1600536814019394/wm5047sup1.cif

Structure factors: contains datablock(s) I. DOI: 10.1107/S1600536814019394/wm5047Isup2.hkl

CCDC reference: 1021407

Additional supporting information:  crystallographic information; 3D view; checkCIF report

## Figures and Tables

**Table 1:** Selected bond lengths (Å) and bond angles (°)

Ag1—O1	2.323 (2)	O1—Ag1—O2^i^	82.45 (7)
Ag1—P1	2.3483 (8)	O1—Ag1—O2^ii^	90.28 (8)
Ag1—O2^i^	2.592 (2)	P1—Ag1—O2^i^	112.26 (5)
Ag1—O2^ii^	2.344 (2)	P1—Ag1—O2^ii^	115.95 (6)
O1—Ag1—P1	148.09 (6)	O2^i^—Ag1—O2^ii^	92.63 (10)

Symmetry codes: (i) −*x* + 1, −*y* + 1, *z*; (ii) −*y* + 1, *x*, −*z* + 2.

**Table 2:** Hydrogen-bond geometry (Å, °)

*D*—H⋯*A*	*D*—H	H⋯*A*	*D*⋯*A*	*D*—H⋯*A*
C13—H13⋯O2^i^	0.93	2.51	3.351 (4)	150

Symmetry code: (i) 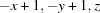.

**Table 3:** Experimental details

Crystal data	
Chemical formula	[Ag_4_(C_9_H_10_O_4_)_2_(C_18_H_15_P)_4_]
*M*_r_	1844.90
Crystal system, space group	Tetragonal, *I* 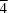
Temperature (K)	105
*a*, *c* (Å)	16.0462 (1), 15.3337 (2)
*V* (Å^3^)	3948.13 (7)
*Z*	2
Radiation type	Mo *K*α
μ (mm^−1^)	1.12
Crystal size (mm)	0.2 × 0.1 × 0.1

Data collection	
Diffractometer	Oxford Gemini S
Absorption correction	Multi-scan (*CrysAlis RED*; Oxford Diffraction, 2006 ▶)
*T*_min_, *T*_max_	0.903, 1.000
No. of measured, independent and observed [*I* > 2σ(*I*)] reflections	21141, 4571, 4425
*R*_int_	0.034
(sin θ/λ)_max_ (Å^−1^)	0.671

Refinement	
*R*[*F* ^2^ > 2σ(*F* ^2^)], *wR*(*F* ^2^), *S*	0.022, 0.048, 1.04
No. of reflections	4571
No. of parameters	240
H-atom treatment	H-atom parameters constrained
Δρ_max_, Δρ_min_ (e Å^−3^)	0.40, −0.52
Absolute structure	Flack *x* determined using 1620 quotients [(*I* ^+^)−(*I* ^−^)]/[(*I* ^+^)+(*I* ^−^)] (Parsons & Flack, 2004 ▶)
Absolute structure parameter	−0.051 (9)

Computer programs: *CrysAlis CCD* and *CrysAlis RED* (Oxford Diffraction, 2006 ▶), *SHELXS2013*, *SHELXL2013* and *SHELXTL* (Sheldrick, 2008 ▶), *ORTEP-3 for Windows* and *WinGX* (Farrugia, 2012 ▶), *DIAMOND* (Brandenburg, 1996 ▶), *publCIF* (Westrip, 2010 ▶) and *PLATON* (Spek, 2009 ▶).

## supplementary crystallographic information

### Crystal data

**Table:**

[Ag_4_(C_9_H_10_O_4_)_2_(C_18_H_15_P)_4_]	*D*_x_ = 1.552 Mg m^−^^3^
*M**_r_* = 1844.90	Mo *K*α radiation, λ = 0.71073 Å
Tetragonal, *I*4	Cell parameters from 14970 reflections
*a* = 16.0462 (1) Å	θ = 3.2–28.4°
*c* = 15.3337 (2) Å	µ = 1.12 mm^−^^1^
*V* = 3948.13 (7) Å^3^	*T* = 105 K
*Z* = 2	Block, colourless
*F*(000) = 1864	0.2 × 0.1 × 0.1 mm

### Data collection

**Table:**

Oxford Gemini S diffractometer	*R*_int_ = 0.034
ω scans	θ_max_ = 28.5°, θ_min_ = 3.1°
Absorption correction: multi-scan (*CrysAlis RED*; Oxford Diffraction, 2006)	*h* = −19→20
*T*_min_ = 0.903, *T*_max_ = 1.000	*k* = −21→21
21141 measured reflections	*l* = −19→20
4571 independent reflections	2 standard reflections every 50 reflections
4425 reflections with *I* > 2σ(*I*)	intensity decay: none

### Refinement

**Table:**

Refinement on *F*^2^	Hydrogen site location: inferred from neighbouring sites
Least-squares matrix: full	H-atom parameters constrained
*R*[*F*^2^ > 2σ(*F*^2^)] = 0.022	*w* = 1/[σ^2^(*F*_o_^2^) + (0.0226*P*)^2^ + 1.5165*P*] where *P* = (*F*_o_^2^ + 2*F*_c_^2^)/3
*wR*(*F*^2^) = 0.048	(Δ/σ)_max_ = 0.001
*S* = 1.04	Δρ_max_ = 0.40 e Å^−^^3^
4571 reflections	Δρ_min_ = −0.52 e Å^−^^3^
240 parameters	Absolute structure: Flack *x* determined using 1620 quotients [(*I*^+^)-(*I*^-^)]/[(*I*^+^)+(*I*^-^)] (Parsons & Flack, 2004)
0 restraints	Absolute structure parameter: −0.051 (9)

### Special details

**Table:**

Geometry. All e.s.d.'s (except the e.s.d. in the dihedral angle between two l.s. planes) are estimated using the full covariance matrix. The cell e.s.d.'s are taken into account individually in the estimation of e.s.d.'s in distances, angles and torsion angles; correlations between e.s.d.'s in cell parameters are only used when they are defined by crystal symmetry. An approximate (isotropic) treatment of cell e.s.d.'s is used for estimating e.s.d.'s involving l.s. planes.
Refinement. Refinement of *F*^2^ against ALL reflections. The weighted *R*-factor *wR* and goodness of fit *S* are based on *F*^2^, conventional *R*-factors *R* are based on *F*, with *F* set to zero for negative *F*^2^. The threshold expression of *F*^2^ > σ(*F*^2^) is used only for calculating *R*-factors(gt) *etc*. and is not relevant to the choice of reflections for refinement. *R*-factors based on *F*^2^ are statistically about twice as large as those based on *F*, and *R*-factors based on ALL data will be even larger.

### Fractional atomic coordinates and isotropic or equivalent isotropic displacement parameters (Å^2^)

**Table:**

	*x*	*y*	*z*	*U*_iso_*/*U*_eq_	
C1	0.42916 (19)	0.52013 (18)	1.13083 (19)	0.0162 (6)	
C2	0.5000	0.5000	1.1958 (3)	0.0156 (8)	
C3	0.4814 (2)	0.4235 (2)	1.2530 (2)	0.0205 (7)	
H3A	0.4329	0.4350	1.2889	0.025*	
H3B	0.4681	0.3765	1.2157	0.025*	
C4	0.5527 (2)	0.4007 (2)	1.3107 (2)	0.0258 (7)	
H4	0.6037	0.3908	1.2837	0.031*	
C5	0.5502 (3)	0.3933 (3)	1.3962 (3)	0.0394 (9)	
H5A	0.5005	0.4026	1.4259	0.047*	
H5B	0.5980	0.3787	1.4269	0.047*	
C6	0.3546 (2)	0.17708 (19)	0.9260 (2)	0.0189 (6)	
C7	0.3733 (2)	0.2094 (2)	0.8438 (2)	0.0252 (7)	
H7	0.4161	0.2480	0.8373	0.030*	
C8	0.3276 (3)	0.1837 (2)	0.7717 (2)	0.0329 (9)	
H8	0.3407	0.2047	0.7168	0.039*	
C9	0.2631 (3)	0.1274 (2)	0.7803 (3)	0.0389 (10)	
H9	0.2332	0.1102	0.7316	0.047*	
C10	0.2434 (2)	0.0970 (2)	0.8615 (3)	0.0346 (9)	
H10	0.1995	0.0597	0.8675	0.042*	
C11	0.2883 (2)	0.1214 (2)	0.9348 (2)	0.0245 (7)	
H11	0.2741	0.1007	0.9894	0.029*	
C12	0.52112 (19)	0.17391 (19)	0.9931 (2)	0.0191 (6)	
C13	0.58867 (19)	0.21305 (19)	1.0339 (2)	0.0230 (6)	
H13	0.5799	0.2591	1.0696	0.028*	
C14	0.6692 (2)	0.1829 (2)	1.0208 (2)	0.0295 (8)	
H14	0.7140	0.2080	1.0489	0.035*	
C15	0.6825 (2)	0.1156 (2)	0.9662 (3)	0.0292 (7)	
H15	0.7361	0.0952	0.9580	0.035*	
C16	0.6160 (2)	0.0786 (2)	0.9240 (2)	0.0267 (7)	
H16	0.6252	0.0339	0.8866	0.032*	
C17	0.5356 (2)	0.1078 (2)	0.9370 (2)	0.0223 (7)	
H17	0.4913	0.0829	0.9079	0.027*	
C18	0.38021 (19)	0.14737 (19)	1.1075 (2)	0.0184 (6)	
C19	0.3973 (2)	0.0620 (2)	1.1117 (2)	0.0233 (7)	
H19	0.4319	0.0376	1.0703	0.028*	
C20	0.3628 (2)	0.0137 (2)	1.1771 (2)	0.0262 (7)	
H20	0.3739	−0.0432	1.1794	0.031*	
C21	0.3121 (2)	0.0500 (2)	1.2389 (2)	0.0258 (7)	
H21	0.2889	0.0174	1.2828	0.031*	
C22	0.2956 (2)	0.1343 (2)	1.2362 (2)	0.0264 (7)	
H22	0.2616	0.1584	1.2784	0.032*	
C23	0.3297 (2)	0.1832 (2)	1.1707 (2)	0.0207 (7)	
H23	0.3187	0.2401	1.1690	0.025*	
O1	0.36944 (13)	0.47026 (13)	1.12166 (14)	0.0186 (5)	
O2	0.43925 (14)	0.58622 (14)	1.08654 (14)	0.0209 (5)	
P1	0.41682 (5)	0.21258 (5)	1.01855 (5)	0.01656 (16)	
Ag1	0.41599 (2)	0.35747 (2)	1.04012 (2)	0.01949 (7)	

### Atomic displacement parameters (Å^2^)

**Table:**

	*U*^11^	*U*^22^	*U*^33^	*U*^12^	*U*^13^	*U*^23^
C1	0.0177 (16)	0.0146 (15)	0.0163 (14)	0.0002 (11)	0.0004 (12)	−0.0030 (11)
C2	0.015 (2)	0.016 (2)	0.0154 (19)	−0.0020 (16)	0.000	0.000
C3	0.0228 (17)	0.0192 (16)	0.0195 (15)	−0.0003 (12)	0.0021 (12)	−0.0004 (12)
C4	0.0282 (19)	0.0209 (17)	0.0284 (18)	0.0034 (13)	−0.0007 (14)	0.0035 (14)
C5	0.050 (3)	0.036 (2)	0.032 (2)	0.0070 (18)	−0.0071 (18)	0.0058 (17)
C6	0.0192 (16)	0.0127 (15)	0.0250 (16)	0.0044 (12)	−0.0039 (12)	−0.0018 (12)
C7	0.0280 (19)	0.0247 (18)	0.0229 (17)	0.0059 (14)	−0.0026 (13)	−0.0032 (13)
C8	0.039 (2)	0.035 (2)	0.0250 (19)	0.0123 (17)	−0.0077 (15)	−0.0018 (16)
C9	0.044 (2)	0.030 (2)	0.042 (2)	0.0116 (17)	−0.0237 (18)	−0.0141 (17)
C10	0.030 (2)	0.0236 (19)	0.050 (2)	0.0006 (15)	−0.0192 (18)	0.0006 (16)
C11	0.0211 (16)	0.0192 (16)	0.0332 (19)	0.0003 (12)	−0.0092 (13)	0.0005 (13)
C12	0.0180 (16)	0.0188 (16)	0.0204 (15)	−0.0011 (12)	−0.0030 (12)	0.0062 (12)
C13	0.0220 (16)	0.0202 (15)	0.0270 (16)	−0.0026 (11)	−0.0015 (14)	0.0025 (14)
C14	0.0206 (17)	0.0303 (19)	0.038 (2)	−0.0044 (14)	−0.0021 (15)	0.0055 (15)
C15	0.0167 (15)	0.0308 (19)	0.040 (2)	0.0026 (12)	0.0062 (16)	0.0078 (17)
C16	0.0252 (18)	0.0231 (18)	0.0318 (18)	0.0028 (14)	0.0071 (14)	0.0032 (14)
C17	0.0214 (16)	0.0205 (16)	0.0250 (18)	−0.0005 (12)	−0.0008 (12)	0.0017 (12)
C18	0.0161 (16)	0.0179 (16)	0.0211 (16)	−0.0015 (11)	−0.0029 (12)	0.0004 (12)
C19	0.0237 (18)	0.0207 (17)	0.0256 (17)	0.0010 (13)	−0.0028 (13)	0.0011 (13)
C20	0.0294 (19)	0.0185 (17)	0.0306 (19)	−0.0010 (13)	−0.0052 (14)	0.0057 (14)
C21	0.0230 (18)	0.0268 (18)	0.0274 (18)	−0.0040 (14)	−0.0028 (14)	0.0097 (14)
C22	0.0217 (19)	0.0320 (19)	0.0255 (18)	0.0024 (14)	0.0021 (14)	0.0041 (14)
C23	0.0191 (17)	0.0198 (17)	0.0234 (17)	0.0030 (12)	−0.0014 (13)	0.0026 (13)
O1	0.0154 (11)	0.0168 (11)	0.0236 (12)	−0.0017 (8)	0.0018 (9)	−0.0032 (9)
O2	0.0212 (12)	0.0188 (11)	0.0225 (12)	−0.0024 (9)	−0.0047 (9)	0.0042 (9)
P1	0.0175 (4)	0.0137 (4)	0.0185 (4)	0.0000 (3)	−0.0015 (3)	0.0002 (3)
Ag1	0.02397 (13)	0.01337 (12)	0.02114 (11)	−0.00192 (9)	0.00038 (10)	0.00018 (9)

### Geometric parameters (Å, º)

**Table:**

C1—O1	1.256 (3)	C13—C14	1.394 (5)
C1—O2	1.270 (4)	C13—H13	0.9300
C1—C2	1.545 (4)	C14—C15	1.383 (5)
C2—C3	1.539 (4)	C14—H14	0.9300
C2—C3^i^	1.539 (4)	C15—C16	1.382 (5)
C2—C1^i^	1.545 (4)	C15—H15	0.9300
C3—C4	1.492 (5)	C16—C17	1.386 (5)
C3—H3A	0.9700	C16—H16	0.9300
C3—H3B	0.9700	C17—H17	0.9300
C4—C5	1.318 (5)	C18—C23	1.387 (5)
C4—H4	0.9300	C18—C19	1.399 (4)
C5—H5A	0.9300	C18—P1	1.817 (3)
C5—H5B	0.9300	C19—C20	1.383 (5)
C6—C7	1.397 (5)	C19—H19	0.9300
C6—C11	1.397 (5)	C20—C21	1.378 (5)
C6—P1	1.825 (3)	C20—H20	0.9300
C7—C8	1.389 (5)	C21—C22	1.380 (5)
C7—H7	0.9300	C21—H21	0.9300
C8—C9	1.380 (6)	C22—C23	1.388 (5)
C8—H8	0.9300	C22—H22	0.9300
C9—C10	1.373 (6)	C23—H23	0.9300
C9—H9	0.9300	Ag1—O1	2.323 (2)
C10—C11	1.391 (5)	Ag1—P1	2.3483 (8)
C10—H10	0.9300	Ag1—O2^i^	2.592 (2)
C11—H11	0.9300	Ag1—O2^ii^	2.344 (2)
C12—C17	1.386 (5)	O2—Ag1^iii^	2.344 (2)
C12—C13	1.400 (4)	O2—Ag1^i^	2.592 (2)
C12—P1	1.827 (3)		
			
O1—C1—O2	124.7 (3)	C15—C14—H14	120.0
O1—C1—C2	120.0 (2)	C13—C14—H14	120.0
O2—C1—C2	115.2 (2)	C16—C15—C14	120.0 (3)
C3—C2—C3^i^	110.4 (4)	C16—C15—H15	120.0
C3—C2—C1^i^	110.11 (16)	C14—C15—H15	120.0
C3^i^—C2—C1^i^	113.04 (17)	C15—C16—C17	120.3 (3)
C3—C2—C1	113.04 (17)	C15—C16—H16	119.8
C3^i^—C2—C1	110.10 (16)	C17—C16—H16	119.8
C1^i^—C2—C1	99.8 (3)	C12—C17—C16	120.3 (3)
C4—C3—C2	112.6 (3)	C12—C17—H17	119.9
C4—C3—H3A	109.1	C16—C17—H17	119.9
C2—C3—H3A	109.1	C23—C18—C19	119.2 (3)
C4—C3—H3B	109.1	C23—C18—P1	118.3 (2)
C2—C3—H3B	109.1	C19—C18—P1	122.4 (2)
H3A—C3—H3B	107.8	C20—C19—C18	120.3 (3)
C5—C4—C3	126.0 (4)	C20—C19—H19	119.9
C5—C4—H4	117.0	C18—C19—H19	119.9
C3—C4—H4	117.0	C21—C20—C19	119.8 (3)
C4—C5—H5A	120.0	C21—C20—H20	120.1
C4—C5—H5B	120.0	C19—C20—H20	120.1
H5A—C5—H5B	120.0	C20—C21—C22	120.5 (3)
C7—C6—C11	119.2 (3)	C20—C21—H21	119.7
C7—C6—P1	118.0 (3)	C22—C21—H21	119.7
C11—C6—P1	122.8 (3)	C21—C22—C23	120.0 (3)
C8—C7—C6	119.7 (3)	C21—C22—H22	120.0
C8—C7—H7	120.2	C23—C22—H22	120.0
C6—C7—H7	120.2	C18—C23—C22	120.1 (3)
C9—C8—C7	120.9 (4)	C18—C23—H23	119.9
C9—C8—H8	119.6	C22—C23—H23	119.9
C7—C8—H8	119.6	C1—O1—Ag1	108.13 (19)
C10—C9—C8	119.5 (3)	C1—O2—Ag1^iii^	111.05 (19)
C10—C9—H9	120.2	C1—O2—Ag1^i^	123.53 (19)
C8—C9—H9	120.2	Ag1^iii^—O2—Ag1^i^	106.22 (8)
C9—C10—C11	120.9 (4)	C18—P1—C6	103.13 (15)
C9—C10—H10	119.6	C18—P1—C12	105.12 (14)
C11—C10—H10	119.6	C6—P1—C12	103.22 (15)
C10—C11—C6	119.8 (3)	C18—P1—Ag1	117.56 (10)
C10—C11—H11	120.1	C6—P1—Ag1	114.54 (10)
C6—C11—H11	120.1	C12—P1—Ag1	111.81 (10)
C17—C12—C13	119.4 (3)	O1—Ag1—P1	148.09 (6)
C17—C12—P1	123.1 (2)	O1—Ag1—O2^i^	82.45 (7)
C13—C12—P1	117.5 (2)	O1—Ag1—O2^ii^	90.28 (8)
C14—C13—C12	119.8 (3)	P1—Ag1—O2^i^	112.26 (5)
C14—C13—H13	120.1	O2^ii^—Ag1—P1	115.95 (6)
C12—C13—H13	120.1	O2^ii^—Ag1—O2^i^	92.63 (10)
C15—C14—C13	120.1 (3)		
			
O1—C1—C2—C3	−8.4 (4)	C19—C20—C21—C22	−0.2 (5)
O2—C1—C2—C3	175.2 (3)	C20—C21—C22—C23	0.3 (5)
O1—C1—C2—C3^i^	−132.4 (3)	C19—C18—C23—C22	−1.0 (5)
O2—C1—C2—C3^i^	51.2 (4)	P1—C18—C23—C22	175.4 (3)
O1—C1—C2—C1^i^	108.5 (3)	C21—C22—C23—C18	0.2 (5)
O2—C1—C2—C1^i^	−67.9 (2)	O2—C1—O1—Ag1	100.7 (3)
C3^i^—C2—C3—C4	−60.5 (2)	C2—C1—O1—Ag1	−75.4 (3)
C1^i^—C2—C3—C4	65.0 (4)	O1—C1—O2—Ag1^iii^	−17.0 (4)
C1—C2—C3—C4	175.6 (2)	C2—C1—O2—Ag1^iii^	159.23 (19)
C2—C3—C4—C5	124.2 (4)	O1—C1—O2—Ag1^i^	−144.9 (2)
C11—C6—C7—C8	−2.2 (5)	C2—C1—O2—Ag1^i^	31.3 (3)
P1—C6—C7—C8	179.7 (3)	C23—C18—P1—C6	−106.6 (3)
C6—C7—C8—C9	1.0 (5)	C19—C18—P1—C6	69.7 (3)
C7—C8—C9—C10	0.5 (6)	C23—C18—P1—C12	145.6 (3)
C8—C9—C10—C11	−0.8 (6)	C19—C18—P1—C12	−38.1 (3)
C9—C10—C11—C6	−0.4 (5)	C23—C18—P1—Ag1	20.5 (3)
C7—C6—C11—C10	1.9 (5)	C19—C18—P1—Ag1	−163.2 (2)
P1—C6—C11—C10	179.9 (3)	C7—C6—P1—C18	−173.8 (2)
C17—C12—C13—C14	−3.0 (5)	C11—C6—P1—C18	8.2 (3)
P1—C12—C13—C14	176.2 (3)	C7—C6—P1—C12	−64.5 (3)
C12—C13—C14—C15	1.4 (5)	C11—C6—P1—C12	117.5 (3)
C13—C14—C15—C16	0.5 (5)	C7—C6—P1—Ag1	57.3 (3)
C14—C15—C16—C17	−1.0 (5)	C11—C6—P1—Ag1	−120.7 (2)
C13—C12—C17—C16	2.5 (5)	C17—C12—P1—C18	85.6 (3)
P1—C12—C17—C16	−176.6 (2)	C13—C12—P1—C18	−93.6 (3)
C15—C16—C17—C12	−0.6 (5)	C17—C12—P1—C6	−22.2 (3)
C23—C18—C19—C20	1.2 (5)	C13—C12—P1—C6	158.7 (2)
P1—C18—C19—C20	−175.1 (3)	C17—C12—P1—Ag1	−145.8 (2)
C18—C19—C20—C21	−0.6 (5)	C13—C12—P1—Ag1	35.1 (3)

Symmetry codes: (i) −*x*+1, −*y*+1, *z*; (ii) −*y*+1, *x*, −*z*+2; (iii) *y*, −*x*+1, −*z*+2.

### Hydrogen-bond geometry (Å, º)

**Table:**

*D*—H···*A*	*D*—H	H···*A*	*D*···*A*	*D*—H···*A*
C13—H13···O2^i^	0.93	2.51	3.351 (4)	150

Symmetry code: (i) −*x*+1, −*y*+1, *z*.
